# Long-Term Prevalence of Fungal Keratitis at a Swiss Tertiary Eye Clinic

**DOI:** 10.3390/microorganisms12081637

**Published:** 2024-08-10

**Authors:** Anahita Bajka, Sadiq Said, Chantal Quiblier, Bettina Schulthess, Ilana Reinhold, Daniel Barthelmes, Sandrine Anne Zweifel, Frank Blaser

**Affiliations:** 1 Department of Ophthalmology, University Hospital Zurich, University of Zurich, 8091 Zurich, Switzerland; anahita.bajka@usz.ch (A.B.);; 2 Institute of Medical Microbiology, University of Zurich, 8006 Zurich, Switzerland; 3 Department of Infectious Diseases and Hospital Epidemiology, University Hospital Zurich, 8091 Zurich, Switzerland

**Keywords:** fungal keratitis, fungal ocular infections, contact lenses, cornea, fungal culture, yeasts, molds, *Aspergillus*, *Candida*, contamination

## Abstract

Fungal keratitis is a rare yet severe infection of the cornea. Fungal species distribution depends on the climate and socioeconomic status and can show regional variation. This retrospective single-center study was conducted at a tertiary eye care center and the collaborating Institute of Medical Microbiology in Switzerland. On investigating all fungal-positive corneal scrapings and contact lens assessments of patients with keratitis from January 2012 to December 2023, 206 patients were identified, of which 113 (54.9%) were female. The median age was 38 (IQR 29.8, [18–93]), and 154 (74.8%) applied contact lenses. The most commonly found pathogen was *Candida* spp., followed by *Fusarium* spp. Molds were 1.8 times more common than yeasts. Linear regression showed no significant increase or decrease in the infection rate over time (*p* = 0.5). In addition, 10 patients (4.9%) were found to have coinfections with *Acanthamoeba*, 11 (5.3%) with HSV-1, none with HSV-2, and 4 (1.9%) with VZV. This study provides a long-term overview of fungal-positive corneal scrapings and contact lens specimens of patients with fungal keratitis. Based on our results, coinfections with *Acanthamoeba*, HSV, and VZV are frequent, especially in patients wearing contact lenses. Thus, wearing contact lenses may facilitate coinfection in fungal keratitis.

## 1. Introduction

Microbial keratitis is a globally prevalent disease estimated to cause up to two million cases of unilateral blindness every year, without accounting for the underreporting in less developed countries [1]. Particularly, fungal keratitis may present a high economic burden due to the high costs of follow-up visits, antifungal medication, and long treatment [2]. Fungal keratitis represents a rare but serious corneal infection [3,4]. Corneal scarring, ulceration, or perforation, with subsequent permanent visual impairment, may occur, even with adequate treatment, underscoring the severity of fungal infections [5,6]. Research has reported various fungi as culprits for microbial keratitis, of which *Fusarium* spp. are most commonly found globally [7].

The disease is more prevalent in rural regions with subtropical or tropical climates and in socioeconomically less fortunate countries [2]. However, fungal keratitis also occurs in developed countries and temperate climate zones, which lie between 23.5° and 66.5° north and south latitudes, respectively [8]. There are various factors that put patients at risk of acquiring such corneal infections. These include risky behaviors, such as wearing contact lenses; patient-specific factors, such as ocular surface disease; local (e.g., applying topical corticosteroids) or systemic immunosuppression; rheumatological disorders and immunodeficiency states; and risk factors, such as post-corneal surgery or ocular trauma, especially with vegetative matter [9,10,11,12].

Patients with fungal keratitis may present with ocular pain, a decrease in vision, photophobia, conjunctival injection, and excessive tearing or secretion [9], whereby the symptom onset may be insidious [3,13]. Clinically, corneal infiltrates in fungal keratitis typically appear whitish to grayish, with feathery margins, and may be accompanied by satellite lesions, which are multilocal foci of infection surrounding the primary infiltrate [13]. In addition to the clinical suspicion on slit-lamp examination, imaging using anterior segment optical coherence tomography (OCT) and in vivo confocal microscopy (IVCM) may be helpful in the diagnosis [14,15]. The latter aims to detect hyphae in molds and pseudohyphae in yeasts [16] and may reveal *Acanthamoeba* coinfection by demonstrating double-walled, hyperreflective, round structures, which represent *Acanthamoeba* cysts [17]. However, the final diagnosis aims to detect the underlying pathogenic fungus, requiring corneal scrapings or corneal biopsy. The invasive corneal sampling procedures and analysis of contact lenses or their storage solution may shorten the time to diagnosis and hence reduce the time to treatment [18]. Identifying the culprit organism and further antifungal susceptibility testing help to treat the patient more efficiently [18,19]. Early antifungal treatment decreases the rate of infectious progression and ocular complications [4,19]. The initial management is often conservative and includes topical antifungal medication, which may be supplemented by systemic antifungal medication and topical antibiotics [18]. Surgical options are applied in severe cases and include corneal cross-linking (photoactivated chromophore for infectious keratitis corneal cross-linking, i.e., PACK-CXL), superficial keratectomy, and penetrating keratoplasty (PKP) [10]. Evisceration is a last-resort procedure in cases with uncontrollable endophthalmitis.

The tertiary care center in this study serves as a national referral center for patients with various microbial keratitis, especially if there is a suspicion of fungal infection. Regarding the mentioned challenges in the early detection and appropriate management of fungal keratitis, this study aims to retrospectively examine corneal fungal infections confirmed by culture or polymerase chain reaction (PCR) and to review the current literature. Further, the aim of this study is to provide an overview of all detected fungi in corneal scraping and contact lens specimens.

## 2. Materials and Methods

### 2.1. Study Design

This was an investigator-initiated, retrospective, single-center study conducted at the Department of Ophthalmology at the University Hospital Zurich in collaboration with the Institute of Medical Microbiology at the University of Zurich, both in Switzerland. All fungal-culture- or PCR-positive test results of corneal scrapings and contact lens specimens between January 2012 and December 2023 were identified, whereby all patients had a clinical presentation of keratitis and had undergone corneal scraping. Contact lens specimens were additionally analyzed if they were available. The local ethics committee signed a declaration of non-responsibility for this project (BASEC-Nr. Req-2023-01146). Still, all data were handled according to Good Clinical Practice guidelines.

### 2.2. Data Collection—Microbiological Data

The data were extracted from the laboratory information system of the Institute of Medical Microbiology. Specimens from patients younger than 18 years of age were excluded from the study. The dataset included corneal scraping and contact lens samples from the Department of Ophthalmology at the University Hospital Zurich, which culturally or molecularly tested positive for yeast or mold. The included materials comprised corneal specimens and contact lenses, whereby only the first isolate of each fungal species per patient and per year was included. Fungal nomenclature is constantly changing due to the more extensive use of molecular technologies and because the dual-species nomenclature (teleomorph/anamorph) was deserted in 2013 [20]. In this study, the names most commonly used in clinical microbiology were used, followed by the names of the current fungal taxonomy according to MycoBank.org in brackets [21]. Furthermore, these cases were investigated for viral or parasitological coinfections confirmed by PCR for herpes simplex virus (HSV)-1 and (HSV)-2, varicella zoster virus (VZV), and *Acanthamoeba*. Lastly, the applied treatment was analyzed.

### 2.3. Identification of Yeast and Mold

The identification of yeast and mold in clinical specimens was performed using standard microbiological techniques at the lab, as described previously [19,22]. Briefly, yeast isolates were identified to the genus or species level by visual differentiation of *Candida* spp. cultivated on BL™ Rice Extract Agar or CHROMagar Candida Medium (BD, Heidelberg, Germany), the API-ID32C^®^ biochemical assay (bioMérieux, Marcy l’Etoile, France), matrix-assisted laser desorption/ionization time-of-flight mass spectrometry (MALDI-TOF MS; MALDI Biotyper, Bruker Daltonics, Bremen, Germany), or sequencing of the internal transcribed spacer (ITS) region (or the 28S ribosomal RNA gene). For mold identification, the clinical specimen was first cultured on general and fungal-selective media at 25 °C for a maximum of 3 weeks, followed by subcultures on different types of media and at incubation temperatures (25–56 °C), depending on genus identification. Phenotypic identification was achieved by macroscopic and microscopic examination according to de Hoog et al. [23]. For molecular identification, chromosomal DNA was isolated using the InstaGene extraction matrix (Bio-Rad Laboratories, Hercules, CA, USA) according to the instructions provided by the manufacturer. The intergenic spacer region (ITS) was then amplified and sequenced. The resulting sequencing data were examined using the ITS SmartGene custom database, adhering to the identification guidelines established by Ciardo et al. [22].

### 2.4. Statistical Analysis

Descriptive statistics were presented as counts and frequencies for categorical data, including information about data distribution in ratios and percentages. To compare ratios over the years, linear regression models were applied, where a *p*-value < 0.05 was considered significant. All evaluations were carried out using the statistical software R (version 4.3.3; R Foundation for Statistical Computing, Vienna, Austria).

## 3. Results

**Figure 1:**
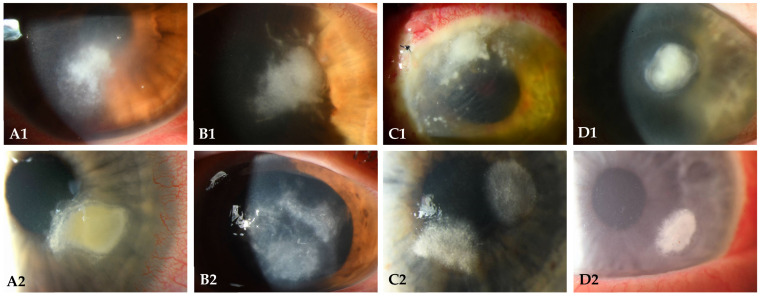
Slit-lamp photography of different patients with fungal keratitis, obtained by direct illumination or scleral scatter. The following fungi are shown: (**A1**,**A2**) *Fusarium solani*, (**B1**) *Aspergillus fumigatus*, (**B2**) *Aspergillus flavus*, (**C1**) *Paecilomyces* sp., (**C2**) *Scedosporium* sp., (**D1**) *Candida albicans*, and (**D2**) *Candida lipolytica*.

From January 2012 to December 2023, all fungal-positive culture or PCR-positive test results of corneal scrapings and/or contact lenses at our institution were assessed. In total, 206 patients were identified, of which 113 (54.9%) were female and 5 (2.4%) patients had bilateral infections. The median age was 38 (interquartile range (IQR) 29.8, range [18–93]). Table 1 provides an overview of the patient demographics. Figure 1 provides examples of clinical findings in fungal keratitis caused by different species.

Of all the 206 patients, 130 (63.1%) patients had positive corneal scraping specimens, of whom 21 (10.2%) patients additionally had positive contact lens specimens. Further, 78 (37.9%) patients had only positive contact lens results, whereby no fungi were detected in corneal scrapings. Regarding contact lens behavior, 154 of the 206 (74.8%) patients wore lenses at symptom onset.

**Figure 2:**
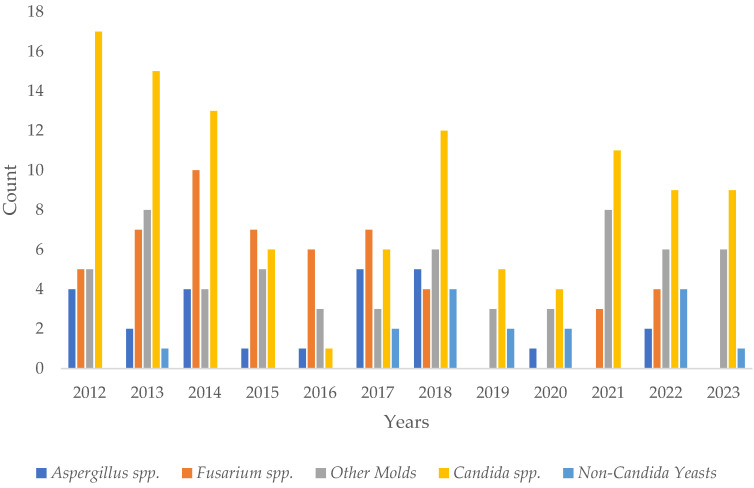
Yearly breakdown of all detected fungi. Each detected species is only counted one time per patient a year.

**Figure 3:**
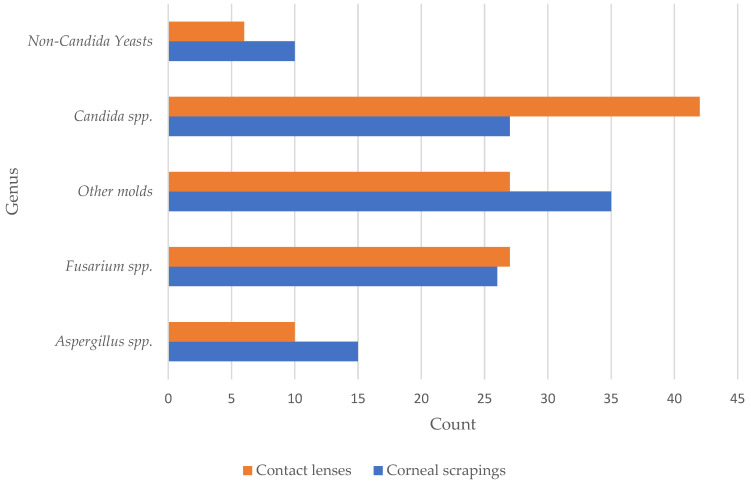
Overview of all detected fungi. Each detected species is only counted one time per patient a year.

The maximum number of annual cases was 33 in 2013, and the minimum number of annual cases was 10 each in 2019 and 2020 (median 22.5, IQR 16.3, [11–33]). Applying linear regression models, an increasing or decreasing trend in fungal corneal infections over time was not found (*p*-value = 0.1). Regarding the number of infections across different seasons, 43 (20.9%) cases were detected during spring, 75 (36.4%) during summer, 51 (24.7%) during autumn, and 37 (18.0%) during winter. Table 2 provides a detailed overview of all detected fungi for each subgroup and further depicts the ratio of molds to yeasts. Overall, the ratio of molds to yeasts was 1.8 to 1. Linear regression over the years showed no significant change in the ratio over time (*p*-value = 0.5). Supplementary Table S1 provides a detailed classification of all detected fungi. Figure 2 provides an overview of all detected subgroups of fungi per year from 2012 to 2023. Figure 3 shows all detected fungi in a grouped manner.

Fungal culture assessments were performed for all patients regardless of the PCR results. In 197 of the 206 (95.6%) patients, the fungal culture was positive, whereas 12 (3.4%) patients had simultaneously positive PCR test results. In nine patients (4.3%), only PCR tests were positive. 

Regarding possible contamination, 14.4% of all detected fungi were species that are more likely to be contamination.

Overall, 25 of the 206 (12.1%) patients had coinfections, whereby 10 (4.9%) were PCR positive for *Acanthamoeba* and 15 (7.3%) patients were PCR positive in the virologic assessments. Further analyzing the last, 11 of the 15 (73.3%) were positive for HSV-1, 4 of the 15 (26.3%) were positive for VZV, and none were positive for HSV-2. All patients with *Acanthamoeba* positivity applied contact lenses. Regarding the PCR-positive virologic results, 8 of the 11 (72.7%) patients with HSV-1 positivity and 3 of the 4 (75%) with VZV positivity used contact lenses. Hence, overall 84% of the patients with coinfections applied contact lenses. Table 3 displays a detailed overview of all coinfections.

Regarding treatment, of all 128 patients with positive corneal scraping tests, 72 (56.3%) patients received only topical antifungal treatment, with natamycin and/or voriconazole and/or amphotericin B eye drops. Of the 128 patients, 3 (2.3%) patients received only systemic antifungal therapy (with voriconazole, fluconazole, posaconazole, or caspofungin intravenously) but combined with topical antibiotics. Of 128 patients, 24 (18.8%) patients received both topical and systemic antifungal therapy. In addition, five (3.9%) cases required surgical intervention, of which three underwent PKP à chaud, one underwent PACK-CXL, and one underwent pars plana vitrectomy (PPV) due to a traumatic perforation of the eye. Regarding the 78 patients with negative corneal scraping results but positive contact lens assessments, 28 (35.9%) patients received topical antifungal treatment, and 2 (2.6%) patients received both topical and systemic antifungal treatment. Table 4 provides an overview of the applied treatment.

## 4. Discussion

This study investigated all positive fungal culture and PCR test results of corneal scrapings and contact lens assessments at a tertiary eye clinic. Over the investigated period of 12 years, 206 patients were identified. At symptom onset, most patients wore contact lenses. The most commonly identified genus of fungi was *Candida* spp., followed by *Fusarium* spp. Comparing all molds against yeasts, molds were detected more commonly, in line with studies performed in warmer climates. For example, Lin et al. [24] reported that more than 60% of all detected fungi were from the *Fusarium* and Asper-gillus species. Several Studies have shown an increase in mold keratitis over the past decades in Europe [25]. Oliveira et al. [26] described a significant increase of molds keratitis in the Netherlands between 2005 and 2016, Olivier et al. [27] an increase of filamentous fungal keratitis in France between 2014 and 2018. Seitz et al. described an increase of molds keratitis in Germany since two years in 2015. Ong et al. [28] detected a contact lens associated increase of filamentary fungal keratitis in the UK between 2014 and 2018, similar to a study by Roth et al. [29] where the fungal keratitis in con-tact lens wearers was typically induced by molds. Therefore, also in temperate climate zones and particularly among contact lens wearers, molds, especially *Fusarium* sp., seem to be a relevant pathogen for acquiring fungal keratitis [29,30,31].

Corneal scrapings are an essential diagnostic tool to confirm suspected fungal keratitis. Our tertiary care center has internally standardized the corneal scraping process, aiming to improve quality. A lid speculum and a sequence of smear preparations specifically depending on the suspected diagnosis are applied, whereby bacterial culture, fungal culture, virologic PCR, and *Acanthamoeba* PCR are examined. In cases of suspected fungal keratitis with negative culture results, panfungal PCR is additionally performed. The latter method is more expensive than standard cultures. Finally, regardless of the invasive diagnostic methods, clinical suspicion remains one of the main pillars in the diagnosis of fungal keratitis. Ophthalmologists should suspect fungal origin in corneal infections with multilocular infiltrates (i.e., satellite lesions) or ones with feathery margins [32].

This study detected coinfections with *Acanthamoeba*, HSV-1, and VZV. However, the distribution was similar between *Acanthamoeba* and HSV-1, with a total of about 15% of all corneal scraping and contact lens assessments performed. Our tertiary eye care center routinely investigates all corneal scrapings and contact lens examinations for bacteria, fungi, parasites, and viruses in any patient with a clinical presentation of infectious keratitis in order to detect and treat coinfections as early as possible. Most patients with coinfections were wearing contact lenses at the time of symptom onset. Contact lens wear is a known risk factor for the development of fungal keratitis but also appears to be a risk factor for the development of co-infection in fungal keratitis [14]. This is not surprising in the context of Acanthamoeba keratitis, as contact lens wear is a well-recognized risk factor for the development of Acanthamoeba keratitis [31]. In the literature, the co-existence of fungi and Acanthamoeba have been previously discussed, based on primary Acan-thamoeba keratitis, where fungi are a co-infection. It is postulated, that an endosymbio-sis of certainn fungi with Acanthamoeba enables the fungi to multiply within the en-capsulated amoeba, allowing them protection from macrophage degradation [32]. In addition, some fungi, such as *Fusarium* sp., may use endosymbiosis not only for their own growth, but also to support excystation of Acanthamoeba [33]. This synergistic trait may facilitate and enhance co-pathogenicity. Nevertheless, there is little data on the incidence of Acanthamoeba co-infections in primary fungal keratitis, as well as for the incidence of herpes virus co-infections in primary fungal keratitis [34,35]. Based on our data, herpes keratitis in contact lens wearers may be a further risk factor for the de-velopment of fungal keratitis. The epithelial defect caused by herpes keratitis, together with contact lens wear, may favor the entry of any fungal elements present into the corneal stroma. Further research is needed to clarify this issue.

Since spurs are ubiquitous, from the moment of taking the sample up to the analysis in the laboratory, samples may be contaminated [29]. Therefore, it remains important to emphasize that each case of possible contamination should be analyzed individually to reduce potential overtreatment, especially regarding antifungal treatment of keratitis when only contact lens assessments come back positive. Considering the possibility of false-positive test results, all laboratory findings must be critically regarded and correlated with the clinical picture. Even common contaminants, such as *Penicillium* sp., *Cladosporium* sp., *Saccharomyces* sp., and *Malassezia* sp., may in fact be the causing organism for the infectious keratitis itself. In order to reduce the rate of contamination, special care must be taken in the corneal scraping process to not touch the swab’s tip, as well as in the laboratory where the final analysis takes place. In this study, the species of fungi that are more commonly associated with contamination were detected in 14.4% of the cases. Comparing this rate with the percentage of treated patients, only 62.6% were treated with antifungal medication. Thus, it remains relevant to mention that each case is also evaluated individually. If the keratitis got better under antibiotic treatment, antifungal treatment was not started, presuming that the detected fungi possibly were a contamination.

Contact lenses and trauma with vegetative matter remain the most important risk factors for acquiring a fungal corneal infection [18]. Yi et al. [36] described the relevance of biofilms in contact lenses associated with fungal keratitis. They postulated that fungi reside within contact lens solutions or lens cases and use the lens as a mediator to reach the cornea by creating a biofilm either on the lens case or on the lens itself, thus evading the overnight lens disinfection [37]. A recent study by Schrenker et al. [38] has shown that polyhexanide-based agents can lack efficacy against *Fusarium* and that Aldox- or hydrogen-peroxide-based storage fluids may reduce the risk of *Fusarium*. Wu et al. [39,40] analyzed the risk factors of contact lens contamination, identifying neglecting to clean the lens case or prolonged use as risk factors. Moreover, hygienic handling of contact lenses helps to avoid corneal infections, as patients failing to wash their hands before the lens application are at greater risk for infectious keratitis [41]. Thus, all patients should receive thorough instructions regarding hygiene measures and handling of the lenses.

In this study, most patients received topical antifungal treatment, a minority received combined topical and systemic treatment, and only a few cases required surgical intervention. This matches the results of other studies [24]. Comparing the subgroups, mainly patients with positive corneal scraping specimens or with positive corneal scraping and positive contact lens specimens were treated, supporting the important role of corneal scrapings compared to contact lens specimens only.

The strengths of this study are the analyzed long-term period from 2012 to 2023 and the broad range of fungi species included. Moreover, this study could show that a lot of patients were wearing contact lenses at symptom onset, including patients with coinfections, such as HSV-1 and VZV. However, this study has several limitations. First, its retrospective nature is inherently prone to selection bias and missing data. Moreover, the single-center design and geographical location may not allow extrapolation of the findings. Furthermore, whether patients had been traveling during the time of infection onset was not investigated. Moreover, confocal microscopy results were not included in this study. Lastly, different types of contact lenses were not subinvestigated. Future research should include a broader diagnostic and treatment workup.

## 5. Conclusions

In summary, this study provides valuable insights regarding culture- and PCR-positive fungal keratitis patients living in a temperate climate. Molds seem to represent the main causing organisms, though an increasing trend of infections over time was not observed. Based on our results, coinfections with *Acanthamoeba*, HSV, and VZV were frequent, especially in patients wearing contact lenses. Further investigations are required to elaborate on this matter.

## Figures and Tables

**Table 1:** Overview of all subgroups. The number of patients and the number of fungal-positive specimens per group is provided, including percentages and ranges with standard deviations in brackets.

Subgroup	Number of Eyes n	Number of Patients n	Age Median (IQR, [Range])	Gender Ratio Female:Male
Corneal scrapings	130 (61.6%)	128 (62.1%)	47.5 (31, [19–93])	75:53
Contact lenses	81 (38.4%)	78 (37.9%)	30.5 (15.8, [18–81])	38:40
Total	211 (100%)	206 (100%)	42.99 (29.8, [18–93])	113:93

**Table 2:** Overview of all fungi species detected from 2012 to 2023 for each subgroup. Each detected species is only counted one time per patient a year. The asterisk (*) marks species that are possibly associated with contamination.

	Corneal Scrapings		Contact Lenses	
Species	n	Percentage (%)	n	Percentage (%)
*Aspergillus flavus*	2	1.8	6	5.5
*Aspergillus fumigatus*	9	8.0	2	1.8
*Aspergillus glaucus*	2	1.8	1	0.9
*Aspergillus niger*	2	1.8	1	0.9
*Aspergillus* spp.	15	13.3	10	9.2
*Fusarium dimerum*	5	4.4	1	0.9
*Fusarium oxysporum*	4	3.5	2	1.8
*Fusarium solani*	1	0.9	1	0.9
*Fusarium* sp.	16	14.2	23	21.1
*Fusarium* spp.	26	23.0	27	24.8
*Acremonium* sp.	8	7.1	3	2.8
*Alternaria* sp.	3	2.7	2	1.8
*Beauveria* sp. *	0	0.0	2	1.8
*Botrytis* sp. *	2	1.8	0	0.0
*Cladosporium* sp.	0	0.0	4	3.7
*Mucor* sp.	0	0.0	1	0.9
*Paecilomyces lilacinus*	2	1.8	1	0.9
*Paecilomyces* sp.	3	2.7	4	3.7
*Penicillium* sp. *	5	4.4	7	6.4
*Phoma* sp.	3	2.7	0	0.0
*Pleosporales* sp. *	1	0.9	0	0.0
*Rhizopus* sp.	1	0.9	0	0.0
*Sarocladium* sp.	1	0.9	0	0.0
*Scedosporium* sp.	5	4.4	0	0.0
*Scopulariopsis* sp. *	0	0.0	1	0.9
*Ulocladium* sp.	1	0.9	0	0.0
Other molds	35	31.0	25	22.9
Total molds	76	67.3	62	56.9
*Candida albicans*	4	3.5	0	0.0
*Candida fabianii*	1	0.9	0	0.0
*Candida famata*	1	0.9	1	0.9
*Candida glabrata*	1	0.9	1	0.9
*Candida guilliermondii*	2	1.8	5	4.6
*Candida lipolytica*	3	2.7	6	5.5
*Candida orthopsilosis*	0	0.0	1	0.9
*Candida parapsilosis*	9	8.0	24	22.0
*Candida pelliculosa (Hansenula anomala)*	0	0.0	1	0.9
*Candida tropicalis*	3	2.7	0	0.0
*Candida* sp.	3	2.7	3	2.8
*Candida* spp.	27	24.0	42	38.5
*Cryptococcus* sp. *	2	1.8	1	0.9
Malassezia restricta *	1	0.9	0	0.0
*Malassezia* sp. *	1	0.9	0	0.0
*Hanseniaspora* sp. *	0	0.0	1	0.9
*Rhodotorula* sp.*	1	0.9	1	0.9
Saccharomycetaceae *	2	1.8	0	0.0
*Saccharomyces* sp. *	3	2.7	2	1.8
Non-*Candida* yeasts	10	8.9	5	4.6
Total yeasts	37	32.8	47	43.1
Total	113	100.0	109	100.0
Ratio of molds to yeasts	2.05 to 1		1.31 to 1	

**Table 3:** Overview of all coinfections, displayed in numbers with percentages and further analyzed regarding contact lens usage. HSV = herpes simplex virus; VZV = varicella zoster virus; N/A = not applicable.

Coinfection	Patients	Contact Lens Wearers
*Acanthamoeba*	10 (4.9%)	10 (100%)
HSV-1	11 (5.3%)	8 (72.7%)
HSV-2	0 (0%)	N/A
VZV	4 (1.9%)	3 (75%)
Total	25 (12.1%)	21 (84%)

**Table 4:** Overview of patients receiving treatment, displayed as number of patients and percentages.

	Patients	Those Who Received Treatment
Corneal scrapings	128 (62.1%)	99 (77.3%)
Contact lenses	78 (37.9%)	30 (38.5%)
Total	206 (100%)	129 (62.6%)

## Data Availability

The original contributions presented in the study are included in the article/Supplementary Materials. Further inquiries can be directed to the corresponding author.

## Supplementary Materials

The following supporting information can be downloaded at https://www.mdpi.com/article/10.3390/microorganisms12081637/s1: Table S1. Overview of all detected types of fungi. In cases where the specific species was not detected, the genus or family is listed.
